# Analytical improvements and developments in stable isotope laboratories for HCNOS analyses

**DOI:** 10.1016/j.mex.2024.102769

**Published:** 2024-05-19

**Authors:** Grzegorz Skrzypek, Philip J.H. Dunn, Gwenaël Imfeld

**Affiliations:** a The University of Western Australia, Crawley, Western Australia, Australia; b National Measurement Laboratory, LGC, London, UK; c Université de Strasbourg, CNRS UMR 7063, Strasbourg F-67084, France

The analyses of the stable isotope compositions of light elements, such as hydrogen (H), carbon (C), nitrogen (N), oxygen (O), and sulphur (S), are now widely employed across various research areas, including agriculture, archology, biochemistry, biology, chemistry, geochemistry, forensics, hydrogeology, medicine, and many others. These techniques, introduced in the 1950′s, initially required intensive effort and used offline preparation methods alongside manually operated dual-inlet isotope ratio mass spectrometers. To enhance accessibility and throughput, technical advancements and automation have become imperative. In the 1980′s, the advent of multiport and semi-automatic dual-inlet systems alleviated the workload and expedited sample processing (e.g., [1,2]). Subsequently, the late 1990′s saw the introduction of continuous-flow systems, substantially reducing analysis time and associated costs, enabling the routine analysis of tens or hundreds of samples overnight (e.g., [3]). Post-2000, further technological improvements, including laser systems as alternatives to traditional magnetic sector Isotope Ratio Mass Spectrometry (IRMS), not only reduced maintenance costs and operational complexities but also complemented the development of peripheral devices (e.g., [4,5]). These devices, including advanced elemental analysers and gas and liquid chromatography systems, facilitate compound-specific stable isotope analyses (e.g., [6]). Currently, a broad range of instruments is available for analysing stable HCNOS isotope compositions across various materials and scales. These include Secondary Ion Mass Spectrometry (SIMS, e.g., CAMECA 1280), Inductively Coupled Plasma Mass Spectrometry (MC-ICP-MS, e.g., Neptune), and Ion Trap Mass Analyser (e.g., Orbitrap Exploris) (e.g., [[7], [8], [9]]). Nevertheless, dual-inlet systems interfaced with traditional magnetic sector IRMS persist as the benchmark analytical reference method and gold standard for reference material calibration (e.g., [10]).

The widespread adoption of stable isotope analyses and the increased availability of mass spectrometers have catalysed interest in new research avenues and spurred the development of previously unexplored analytical techniques. Numerous laboratories and research groups have refined their preparation and measurement methodologies to such an extent that standardisation of methods and interlaboratory cross-validation have become imperative. This necessitates the comprehensive publication of technical details pertaining to preparation and measurement methods. MethodsX journal serves as a platform for the dissemination of laboratory protocols, standard operating procedures, and technical notes, facilitating their implementation in various laboratory settings. By providing accessible and detailed methodologies, MethodsX streamlines the adoption of these techniques across laboratories and word-wide analytical procedure standardisation.

The current Virtual Special Issue of MethodsX is dedicated to recent advancements in stable HCNOS isotope methodologies. It focuses on new protocols, improvement of preparation techniques and measurement quality, and innovation in data processing. For further information, please refer to the following link: https://www.sciencedirect.com/journal/methodsx/special-issue/10QSVVJ7ZVH.

All improvements in analytical methods contribute to the attainment of more robust, accurate, and precise measurements and the generation of higher-quality datasets. Yet, to ensure full data verifiability and comparability, as well as to reduce biases across laboratories or over time within a single laboratory, the standardisation of reporting stable isotope results is of critical importance. In light of this, we strongly advocate for authors and reviewers to adhere closely to the guidelines established by the International Union of Pure and Applied Chemistry (IUPAC) [11]. These guidelines stipulate minimum requirements for the publication of stable HCNOS results, thereby fostering consistency and facilitating meaningful comparisons across studies now and in the future. By embracing IUPAC guidelines, researchers can promote transparency, reliability, and reproducibility in the reporting of stable isotope data and avoid hidden biases in reporting isotope delta results [12].
